# Prevalence and Patterns of Sleep-Disordered Breathing in Indian Heart Failure Population

**DOI:** 10.1155/2021/9978906

**Published:** 2021-07-03

**Authors:** Sajit Kishan, Mugula Sudhakar Rao, Padmakumar Ramachandran, Tom Devasia, Jyothi Samanth

**Affiliations:** ^1^Department of Cardiology, Unity Hospital, Mangalore, Karnataka, India; ^2^Department of Cardiology, Kasturba Medical College Manipal, Manipal Academy of Higher Education (MAHE), Karnataka, India; ^3^Department of Cardiovascular Technology, Manipal College of Health Professions, Manipal Academy of Higher Education (MAHE), Karnataka, India

## Abstract

**Background:**

Sleep-disordered breathing (SDB) is a common yet a largely underdiagnosed entity in developing countries. It is one treatable condition that is known to adversely affect the mortality and morbidity in heart failure (HF). This study is one of the first attempts aimed at studying SDB in chronic HF patients from an Indian subcontinent.

**Objectives:**

The aim of this study was to study the prevalence, type, and characteristics of SDB in chronic HF patients and their association with HF severity and left ventricular (LV) systolic function and also to determine the relevance of SDB symptoms and screening questionnaires such as the Epworth Sleepiness Scale (ESS), Berlins questionnaire, and STOP-BANG score in predicting SDB in chronic HF patients.

**Methods:**

We enrolled 103 chronic heart failure patients aged more than 18 years. Patients with a history of SDB and recent acute coronary syndrome within 3 months were excluded. Relevant clinical data, anthropometric measures, echocardiographic parameters, and sleep apnea questionnaires were collected, and all patients underwent the overnight type 3 sleep study.

**Results:**

The overall prevalence of SDB in our study was high at 81.55% (84/103), with a predominant type of SDB being obstructive sleep apnea (59.2%). The occurrence of SDB was significantly associated with the male gender (*p* = 0.002) and higher body mass index (BMI) values (*p* = 0.01). SDB symptoms and questionnaires like ESS, STOP-BANG, and Berlins also did not have a significant association with the occurrence of SDB in HF patients.

**Conclusions:**

Our study showed a high prevalence of occult SDB predominantly OSA, in chronic HF patients. We advocate routine screening for occult SDB in HF patients.

## 1. Background

Despite advancements in treatment modalities, heart failure is highly prevalent and is still associated with excess mortality and morbidity, particularly in the elderly. Multiple factors may be contributing to the progressive declining course of heart failure. Therefore, recognizing these treatable conditions that may contribute to the progression of heart failure is important.

One of the known causes is severe nocturnal hypoxemia caused by sleep-disordered breathing (SDB), a common underlying condition in HF patients. Compared to the general population, SDB has a very high prevalence of around 50–80% in HF patients [1, 2]. Both SDB and HF are common diseases in the general population which affect around 10% [3] and 2% [4] of them, respectively.

Due to poor awareness of the condition and lack of access to a simple, accurate screening test especially in developing countries like India, many patients are often not diagnosed of SDB. Also, HF patients with SDB are known to comparatively lack symptoms compared to general SDB patients, particularly excessive daytime sleepiness, which could lead to the lack of recognition and detection of SDB in HF patients [5–9].

SDB has equally high prevalence in both the types of HF, i.e., HF with reduced ejection fraction (HFrEF) and HF with preserved ejection fraction (HFrEF), and it goes even more higher from 44% to 97%, in acute decompensated HF patients [10, 11]. Prevalence of OSA in chronic HF patients ranges from 20 to 45% [12, 13]. The presence of OSA itself is associated with a bad prognosis in HF patients [5], though on optimal medical therapy [14]. The prevalence of OSA is higher in HFpEF patients at 69–81%. Similarly, OSA is the predominant type of SDB [12, 15]. The aim of our study was to determine the prevalence, type, and characteristics of SDB in chronic HF patients and association with heart failure severity and left ventricular systolic function.

Further, we also wanted to determine the relevance of SDB symptoms and standard SDB screening questionnaires (Epworth Sleepiness Scale (ESS), Berlins questionnaire, and STOP-BANG score) in predicting SDB in chronic HF patients.

## 2. Methods

This was single-center, cross-sectional, observational study done over a period of 1 year which enrolled 103 chronic HF in-patients. The study was approved by the Institutional Ethics Committee of Kasturba Medical College, Manipal (IEC 78/2019), and performed in accordance with principles of the Declaration of Helsinki. Written informed consents were obtained from each patient before enrollment in the study.

Inclusion criteria were patients aged > 18 years and chronic heart failure (diagnosed as per Framingham criteria, with at least 3 months duration). In-hospital patients who have recovered from an acute decompensation, patients with recent acute coronary syndrome (ACS) in the last 3 months, pregnant women, and previously diagnosed SDB patients on therapy were excluded from the study. Data including clinical history, symptoms, comorbidities, anthropometric measures, and sleep apnea questionnaires (ESS, Berlin questionnaire, and STOP BANG) were collected. Excessive daytime sleepiness was assessed as per patient symptomatology as well as scoring based on the Epworth Sleepiness Scale (ESS). Echocardiography was used to determine the left ventricular ejection fraction (LVEF) and patients classified into 2 groups as per the 2013 ACCF/AHA Heart Failure Guidelines [16], into the heart failure preserved ejection fraction (HFpEF) if LVEF > 40% and heart failure reduced ejection fraction (HFrEF) if LVEF ≤ 40%.

**Figure 1:**
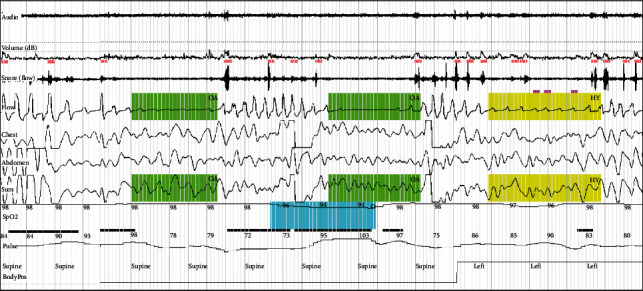
A polygraphy recording showing obstructive apnea events (in green), hypopnea events (in yellow), and desaturation events (in blue).

**Figure 2:**
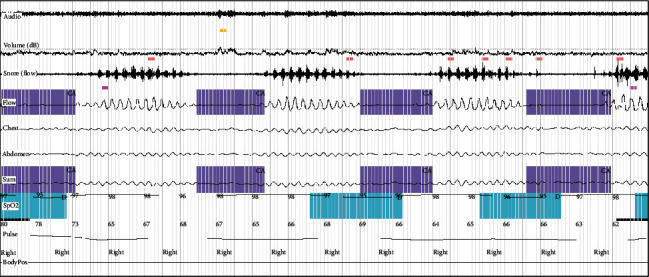
A polygraphy recording (10 epochs) showing central apnea events with Cheyne-Stokes respiration (in purple).

All study patients were subjected to an overnight type 3 sleep study using MediByte®, a 12-channel home sleep study machine which recorded Snoring events, oronasal pressure airflow, thermal airflow, chest/abdominal/sum efforts, oxygen saturation, pulse rate, body position, and user events. The study was analyzed, and the various desaturation events and respiratory events such as obstructive, central, mixed apneas, and hypopneas were identified in each epoch manually. Scoring was done to determine various indices such as the apnea hypopnea index (AHI) and oxygen desaturation index (ODI) (Figures 1 and 2).

A diagnosis of SDB was made when AHI is ≥5 respiratory events/hr. SDB were classified either as central sleep apnea (CSA) or obstructive sleep apnea (OSA) as per guidelines [17]. The prevalence and severity of SDB including both CSA and OSA separately were determined in the study population.

SDB severity was compared with the type of HF, LV function (LVEF), heart failure severity class based on NYHA, and NTProBNP levels. Excessive daytime sleepiness symptom and ESS scores were compared with occurrence of SDB. Other screening questionnaires (Berlin questionnaire and STOP BANG) were compared with the occurrence of SDB. The correlation between AHI and screening scores (ESS and STOP-BANG) was determined to look for the role of screening questionnaires in predicting SDB in heart failure patients.

### 2.1. Statistical Method

Data was entered in Microsoft Excel and converted to SPSS vers.21.0 for the analysis. Categorical variables were expressed in frequency and the percentage and continuous variable were expressed in mean and standard deviation. The median and interquartile range were calculated when variables were not normally distributed. Univariate analysis by the linear regression model was done to see the association between the STOP-BANG score and AHI. Data obtained among SDB and non-SDB groups were analyzed using chi-square and multivariable analyses (multivariate binary logistic regression). The ANOVA test was used to see the association between LVEF and stages of SDB.

## 3. Results

### 3.1. Baseline Characteristics

Our study was done on 103 chronic HF patients of 63 males (61.1%) and 40 females (38.9%). The mean age of the study group was 62.65 ± 11.8 yrs. Mean LVEF was 44.2 ± 16%. 44.6% patients had HFpEF and 55.4% had HFrEF. The mean BMI in SDB patients and that in non-SDB patients were 26.6 ± 5.76 and 23.82 ± 4.7, respectively, whereas those in patients with HFrEF and HFpEF were 24.9 ± 4.54 and 27.73 ± 6.64, respectively. The median time since diagnosis of HF was 9 months (interquartile range 6–24). 57.2% patients were in NYHA class II, and 32% patients were class III. The predominant etiology of HF was ischemic heart disease in 71.8% of patients. Fatigue was the most common symptom found in 48 patients (46.66%) followed by snoring in 38 patients (36.9%) (Table 1).

The overall prevalence of SDB in our study was high at 81.5% (84/103), with a predominant type being OSA in 59.2% (61/103). The prevalence of CSA was 22.33% (23/103). The Cheyne-Stokes respiration (CSR) pattern was noted in 3 cases. The occurrence of SDB was significantly associated with the male gender (*p* = 0.002) and higher BMI values (*p* = 0.01). The mean age in SDB patients and that in non-SDB patients were 62.93 ± 11.89 and 61.33 ± 12.11, respectively (*p* = 0.615).

Severity of SDB was graded based on AHI; according to which, 24 patients (23.3%) had mild SDB, 18 patients (17.4%) had moderate SDB, and 42 patients (40.7%) had severe SDB. Out of the 61 patients with OSA, 20, 14, and 27 patients had mild, moderate, and severe OSA, respectively. Likewise, out of the 23 patients with CSA, 4, 3, and 16 had mild, moderate, and severe CSA, respectively. All 3 patients with CSR had severe SDB.

It was found that 38/45 (84.4%) of HFpEF pts and 46/58 (79.3%) of HFrEF pts had SDB. 28 and 10 out of 38 patients with HFpEF patients had OSA and CSA, respectively, whereas 33 and 13 out of 46 patients with HFrEF patients had OSA and CSA, respectively. OSA was the predominant type of SDB in both the HFrEF and HFpEF groups (Table 2). There was no significant difference in LVEF between the two groups with the mean LVEF in SDB patients and non-SDB patients being 43.75 ± 16.16 and 46.39 ± 15, respectively (*p* = 0.528). There was no significant difference noted in mean LVEF among various stages of SDB with mean values being 47.96 ± 14.85, 40.44 ± 16.38, and 42.90 ± 16.83 in stages 1, 2, and 3, respectively (*p* = 0.429). There was no significant association between severity of SDB and NYHA class (*p* = 0.88).

Lesser patients reported daytime sleepiness by symptom assessment than by ESS scoring. Based on ESS scoring, patients were classified into 2 groups with ESS < 10 and ≥10. 57.6% of SDB patients had ESS < 10 and 42.4% had ESS ≥ 10. Neither EDS nor a higher ESS score was associated with a higher occurrence of SDB. Also, there was no significant correlation between ESS and AHI (*R* = −0.03 and *p* = 0.76).

We studied SDB screening questionnaires such as STOP BANG and Berlin. Patients were divided based on STOP BANG scores into low (0–2), intermediate (3, 4), and high risk (5–8) categories. Although a statistical association could not be determined, all 13 patients with high-risk STOP BANG scores had SDB predominantly OSA (92.3%). Most of the heart failure patients with SDB had intermediate scores. Also, STOP BANG scores and AHI scores showed weak positive correlation (*R* = 0.19 and *p* = 0.054). The Berlin questionnaire divided the patients into high-risk and low-risk groups. In the high-risk group, 33 (76.7%) out of 43 patients had SDB whereas in the low-risk group, 51 (85%) out of 60 patients had SDB. This shows a higher prevalence of SDB in the low-risk group. However, the association was not statistically significant. OSA was predominant in diabetic patients of our study. However, the association was not significant with diabetes and the occurrence of SDB. A multivariate logistic regression analysis was done to see the predictors of SDB, and we found that the male gender and higher BMI values were independently associated with the occurrence of SDB (Table 3).

## 4. Discussion

Our study included 103 in-patients with chronic heart failure admitted in the tertiary care center in semiurban South India. This study represents one of the first attempts till date to look for SDB prevalence in Indian heart failure patients.

It enrolled heart failure patients, who have been hospitalized and have recovered from an acute decompensation of heart failure, irrespective of the SDB symptom status. Large-scale studies done by Schulz et al. and Arzt et al. also enrolled patients irrespective of SDB symptoms to see the prevalence of occult SDB [2, 11]. Our study group had a male preponderance (61.1%) but with considerable female representation (38.9%). Most studies had similar male preponderance [1, 15]. Etiology of HF in our study group was predominantly ischemic (71.8%) as in most heart failure populations such as in the Trivandrum HF Registry (THFR) where ischemic heart disease cases constituted 72% [18]. In our study, patients did not have the conventional symptoms of SDB as observed in general population [6]. Most of our patients had SDB symptoms that were common to heart failure such as weight gain and fatigue. Weight gain was a major symptom, probably perceived so due to fluid retention and associated recent decompensation. In the multicenter study by Arzt et al., symptoms of nocturnal dyspnea and nocturia were more common [11].

Our study found a very high prevalence (81.55%) of clinically unsuspected SDB in chronic heart failure patients. This prevalence of SDB in chronic heart failure was found to be higher than in the general population. Various similar studies on chronic HF reported a prevalence rate ranging from 50 to 80% [1, 2]. An 86-patient study with stable HF done by Isakson et al. showed the prevalence rate of 85% [19]. Conversely, a large multicenter study from the German SchlaHF registry [11] and also a 218-patient study by Yumino et al. reported a lower prevalence of 46% and 47%, respectively [20]. In a study of 450 patients with stable chronic HF done by Sin et al., SDB prevalence was found to be 61% [21].

In our study, OSA was the predominant type (59.2%) of SDB. Major studies by Schulz et al., Herrscher et al., and Paulino et al. showed similar predominance of OSA in HF patients [2, 12, 22]. Conversely, various other studies showed either similar proportions of both OSA and CSA [1, 23] or a higher prevalence of CSA over OSA [24–26]. The predominance of OSA in our study could be due to differences in patients' characteristics of the Indian population. CSA, however, is more common in HF patients especially in patients with acute heart failure with prevalence ranging from 44% to as high as 97% in various studies [27, 28]. Also, Cheyne-Stokes respiration is known to occur predominantly in acute decompensated HF but was also found in our study which had fairly stabilized HF patients.

Our study had a fairly equal distribution of severity of SDB patients with slight preponderance of severe SDB patients at 40% prevalence. The large SchlaHF registry had predominantly severe forms of SDB in their study of stable chronic HF patients [11]. Similar to the study by Paulino et al. [22], in our study, CSA patients had a predominantly severe type (69.5%) of SDB.

Similarly, it has been documented in various studies that prevalence rates are fairly similar in both HFrEF and HFpEF. OSA was a predominant type in both HFpEF and HFrEF patients. Our study differed in that OSA was a more common type in HFrEF patients [12, 15]. The use of beta blockers in HFrEF patients could have contributed to the decreased prevalence of CSA, like studies done by Paulino et al. and Schulz et al. which have shown that beta blocker usage could improve myocardial function and thereby reduce CSA [29, 30].

LVEF had no significant association with the severity of SDB. Schulz et al. [2] and Javaheri et al. [7] showed that more severely reduced LVEF was associated with an increased risk to develop SDB predominantly CSA. However, a large study by Arzt et al. showed that LVEF did not show a significant association with SDB severity [11]. SDB severity was not associated (*p* = 0.88) with the NYHA class similar to the study by Schulz et al. [2]. Arzt et al. showed that higher AHI values were associated with a higher NYHA functional class [11].

There was no statistically significant correlation between the occurrence of SDB and age. Multivariate analysis showed significant association between SDB and male sex (*p* = 0.002), though this analysis could be influenced by predominantly OSA patients. Studies done by Arzt et al. [11], Schulz et al. [2], and Paulino et al. [22] showed that SDB prevalence increased with older age and male sex. Sin et al. [21] showed age to be an independent predictor of OSA in females only, whereas Yumino et al. [20] showed similar association for both sexes.

Our study did not show significant correlation between the Epworth Sleepiness Scale (ESS) and the occurrence of SDB. Unlike in the general population, SDB patients with heart failure had lower ESS scores and lesser patients reported symptoms of excessive daytime sleepiness. Various studies in HF patients such as that by Herrscher et al. [12] have shown that SDB patients did not evaluate themselves as sleepy according to the ESS, and thus, ESS is becoming a weak instrument to measure sleepiness in these patients [6, 31]. This is in contrast to the general population where excessive daytime sleepiness (EDS) and also the ESS score were important SDB predictors [32]. One possible explanation for the absence of EDS in HF patients with SDB is the increased SNS activity in HF patients than in healthy subjects with the presence counteracting the effects of sleep fragmentation and deprivation [33, 34].

Also, other SDB symptoms did not correlate with the occurrence of SDB in our study. Various studies have shown that patients with HF may not show typical SDB symptoms unlike non-HF patients. Our study showed poor utility of screening questionnaires like STOP BANG and Berlin questionnaire in heart failure patients possibly due to the lack of typical symptoms and risk factors in these patients. No prior studies were done previously in these patient populations. There was higher prevalence of SDB in obese patients with a significant association between SDB and BMI values. However, the multivariate analysis could be influenced by the preponderance of OSA patients. In the study by Schulz et al. [2], AHI was independently associated with BMI and SDB of Obesity. Arzt et al. [6] reported that HF patients have lower BMI for any AHI value, as compared to the general population where AHI increases with an increase in BMI. Apart from obesity, factors such as nocturnal rostral fluid displacement are likely to play a major role in the pathogenesis of OSA in the heart failure population.

There are few limitations to our study. This study was done on a relatively small number of patients, due to resource limitations. As this was also a single-center study, there is a possibility of selection bias. As this study was done on hospitalized patients, the findings cannot be extrapolated to out-patients. SDB was diagnosed in our study by the type III unattended sleep study and not by the gold standard type I attended sleep study. The lack of electroencephalographic (EEG) channels could have led to an underestimation of SDB severity, as the AHI is calculated using recording duration rather than total sleep time. This factor may be crucial, as HF patients are known to have poor sleep quality, with sleep fragmentation and an increase in waking after sleep onset, secondary either to SDB or to periodic limb movements or due to the CHF itself.

Also, there might be some discrepancies regarding the differentiation between obstructive and central events, as differentiation is not with esophageal manometry which is the gold standard as it is not feasible and contraindicated in HF patients. Also, as the sleep study has been scored by a single personnel, there may be interobserver variation which has not been addressed.

## 5. Conclusions

Our study has shown a very high prevalence of occult SDB in chronic heart failure patients compared to other Western studies. Heart failure patients with SDB differ from the nonheart failure SDB patients in symptomatology and clinical presentation. Also, standard SDB screening questionnaires have a poor utility in predicting SDB in heart failure patients. Since there was a high prevalence of OSA in our study, a need for its routine screening in chronic heart failure patients irrespective of symptoms may have to be considered. To our knowledge, this was one of the first attempts aimed at studying SDB in chronic HF patients from the Indian subcontinent.

## Figures and Tables

**Table 1:** Demographic and clinical characteristics of study population (*n* = 103).

		SDB present, *n* = 84 (%)	No SDB, *n* = 19 (%)	*p* value (*χ*^2^)
Gender	Male sex	57 (67.9%)	6 (31.6%)	*0.015*
HF type	HFREF	46 (54.8%)	11 (57.9%)	0.797
	HFPEF	38 (45.2%)	8 (42.1%)	
NYHA	II	48 (57.1%)	11 (57.9%)	0.498
	III	28 (33.3%)	5 (26.3%)	
	IV	7 (8.3%)	2 (10.5%)	
Etiology	Ischemic type	63 (75.0%)	11 (57.9%)	0.265
Drugs	BB	27 (32.1%)	3 (15.8%)	0.261
	ACEI/ARB	14 (16.7%)	3 (15.8%)	1.00
	Diuretics	51 (60.0%)	12 (63.1%)	0.791
	MRA	21 (25.0%)	3 (15.8%)	0.555
	ARNI	8 (9.5%)	2 (10.5%)	0.699
Comorbidities	T2DM	52 (61.9%)	9 (47.4%)	0.268
	HTN	46 (54.8%)	7 (36.8%)	0.303
	Dyslipidemia	30 (35.7%)	3 (15.8%)	0.166
	Hypothyroidism	10 (11.9%)	2 (10.5%)	1.00
	CKD	8 (9.5%)	1 (5.3%)	0.699
	AF	7 (8.3%)	4 (21.1%)	0.098
Symptoms	Snoring	27 (32.1%)	11 (57.9%)	0.06
	SOB during sleep	24 (28.6%)	7 (36.8%)	0.265
	Daytime Sleepiness	28 (33.3%)	7 (36.8%)	0.785
ESS	<10	36 (42.9%)	4 (22.1%)	0.182
	≥10	48 (57.1%)	15 (78.9%)	
STOP BANG risk	Low risk	27 (32.1%)	6 (31.6%)	0.184
	Intermediate risk	45 (53.6%)	12 (63.2%)	
	High risk	13 (15.5%)	0 (0%)	
Berlin Q	High risk	33 (39.3%)	10 (52.6%)	0.293
	Low risk	52 (61.9%)	8 (42.1%)	

SDB: sleep-disordered breathing; HFREF: heart failure with reduced ejection fraction; HFpEF: heart failure with preserved ejection fraction; NYHA: New York Heart Association; BB: beta blockers; ACEI: angiotensin converting enzyme inhibitors; ARB: angiotensin receptor blockers; MRA: mineralocorticoid receptor antagonists; ARNI: angiotensin receptor neprilysin inhibitors; T2DM: type 2 diabetes mellitus; HTN: hypertension; CKD: chronic kidney disease; AF: atrial fibrillation; SOB: shortness of breath; ESS: Epworth Sleepiness Scale; Q: questionnaire.

**Table 2:** Demographic and clinical characteristics between HFrEF and HFpEF.

		HFREF, *n* = 57 (%)	HFPE, *n* = 46 (%)	*p* value (*χ*^2^)
Gender	Male sex	36 (63.15%)	27 (58.69%)	1.00
NYHA	II	29 (50.87%)	32 (69.56%)	0.04
	III	21 (36.84%)	12 (26.08%)	
	IV	7 (12.28%)	2 (4.34%)	
Etiology	Ischemic type	44 (77.19%)	30 (65.21%)	0.512
Drugs	BB	16 (28.07%)	14 (30.43%)	0.664
	ACEI/ARB	10 (17.54%)	7 (15.21%)	1.00
	Diuretics	44 (77.19%)	19 (41.30%)	0.002
	MRA	23 (40.35%)	1 (2.17%)	<0.001
	ARNI	9 (15.78%)	0 (0%)	0.009
Comorbidities	T2DM	37 (64.91%)	24 (52.17%)	0.425
	HTN	24 (42.1%)	29 (63.04%)	0.017
	Dyslipidemia	17 (29.82%)	16 (34.78%)	0.398
	Hypothyroidism	7 (12.28%)	5 (10.86%)	1.00
	CKD	7 (12.28%)	2 (4.34%)	0.294
	AF	4 (7.01%)	7 (15.21%)	0.198
Symptoms	Snoring	16 (28.07%)	22 (47.82%)	0.02
	SOB during sleep	15 (26.31%)	16 (34.78%)	0.280
	Daytime sleepiness	18 (31.57%)	17 (36.95%)	0.408
ESS	<10	21 (36.84%)	19 (41.30%)	0.838
	≥10	36 (63.15%)	27 (58.69%)	
STOP BANG risk	Low risk	26 (45.61%)	7 (15.21%)	0.006
	Intermediate risk	26 (45.61%)	31 (67.39%)	
	High risk	5 (8.77%)	8 (17.39%)	
Berlin Q	High risk	20 (35.08%)	23 (50%)	0.072
	Low risk	37 (64.91%)	23 (50%)	

HFREF: heart failure with reduced ejection fraction; HFpEF: heart failure with preserved ejection fraction; NYHA: New York Heart Association; BB: beta blockers; ACEI: angiotensin converting enzyme inhibitors; ARB: angiotensin receptor blockers; MRA: mineralocorticoid receptor antagonists; ARNI: angiotensin receptor neprilysin inhibitors; T2DM: type 2 diabetes mellitus; HTN: hypertension; CKD: chronic kidney disease; AF: atrial fibrillation; SOB: shortness of breath; ESS: Epworth Sleepiness Scale; Q: questionnaire.

**Table 3:** Predictors of sleep-disordered breathing multivariate analysis.

	OR	95% CI		*p* value
		Lower	Upper	
Male gender	7.571	2.069	27.701	*0.002*
Age	1.027	0.982	1.075	0.245
BMI	1.177	1.024	1.354	*0.022*
NYHA class 1	0.350	0.003	37.572	0.660
NYHA class 2	1.442	0.208	10.009	0.711
NYHA class 3/4	1.580	0.200	12.511	0.665
LVEF	0.988	0.951	1.026	0.534
AF	0.284	0.048	1.691	0.167

OR: odds ratio; CI: confidence interval: BMI: body mass index; NYHA: New York Heart Association; LVEF: left ventricular ejection fraction; AF: atrial fibrillation.

## Data Availability

Data availability will be made whenever asked for.

## Abbreviations

SDB: Sleep-disordered breathing
ESS: Epworth Sleepiness Scale
EDS: Excessive daytime sleepiness
OSAS: Obstructive sleep apnea syndrome
CSA: Central sleep apnea
CSR: Cheyne-Stokes respiration
SHHS: Sleep Heart Health Study
PSG: Polysomnography
AHI: Apnea hypopnea index
SNS: Sympathetic nervous system
RDI: Respiratory disturbance index
ODI: Oxygen desaturation index
NC: Neck circumference.
